# Understanding Centenarians' Psychosocial Dynamics and Their Contributions to Health and Quality of Life

**DOI:** 10.1155/2010/680657

**Published:** 2010-09-26

**Authors:** Leonard W. Poon, Peter Martin, Alex Bishop, Jinmyoung Cho, Grace da Rosa, Neha Deshpande, Robert Hensley, Maurice MacDonald, Jennifer Margrett, G. Kevin Randall, John L. Woodard, L. Stephen Miller

**Affiliations:** ^1^Institute of Gerontology, University of Georgia, GA 30602, USA; ^2^Gerontology Program, Iowa State University, IA 50011, USA; ^3^Human Development & Family Science, Oklahoma State University, OK 74078, USA; ^4^Psychology and Sociology, College of Saint Scholastica, MN 55811, USA; ^5^Family Studies and Human Services, Kansas State University, KS 66506, USA; ^6^Family and Consumer Science, Bradley University, IL 61625, USA; ^7^Psychology, Wayne State University, MI 48202, USA

## Abstract

While it is understood that longevity and health are influenced by complex interactions among biological, psychological, and sociological factors, there is a general lack of understanding on how psychosocial factors impact longevity, health, and quality of life among the oldest old. One of the reasons for this paradox is that the amount of funded research on aging in the US is significantly larger in the biomedical compared to psychosocial domains. The goals of this paper are to highlight recent data to demonstrate the impact of four pertinent psychosocial domains on health and quality of life of the oldest old and supplement recommendations of the 2001 NIA Panel on Longevity for future research. The four domains highlighted in this paper are (1) demographics, life events, and personal history, (2) personality, (3) cognition, and (4) socioeconomic resources and support systems.

## 1. Introduction

A recent review of centenarian research [1] shows a *biopsychosocial* approach that takes into account biological, psychological, and sociological mechanisms and their interactions may be most efficient to understand the multidimensional aspects of extreme longevity. A cursory search through PUBMED [2] on the number of publications in the last five years (2004–2009) in the study of longevity shows that biopsychosocial studies are rare. In fact, in the last five years, no study was noted by PUBMED on either extreme longevity or longevity using biopsychosocial approaches. When specific key words were employed in the search within the biomedical and psychosocial domains and longevity between 2004 and 2009, the ratio of biomedical to psychosocial studies ranged from a low of about 3 : 1 to a high of about 7 : 1. This contradictory situation might reflect the disproportional amount of funding on biomedical compared to psychosocial aspects of aging as evidenced by funding at the US National Institute on Aging [3] and perhaps a general lack of understanding by the research community of the impact and measurement of psychosocial factors among the oldest old. 

In 2001, the NIA convened a panel of clinical, demographic, epidemiologic, and genetics research experts—*NIA Panel on the characterization of participants in studies of exceptional survival in humans*—to provide guidelines on measures that are pertinent in longevity studies and could provide precise information on the “survival” phenotype [4]. The goal of the panel was to develop a set of standard measures that could be used in all studies of exceptional survival. Recommendations were made on the measurement of age, sociodemographic characteristics, health, functional status, psychological and social characteristics, group survival characteristics, data reporting, and availability. Since 2001, a significant amount of information on psychosocial parameters has been discovered to impact longevity and health. One of the goals of this paper is to supplement the 2001 recommendations from the psychosocial domains with recent empirical findings.

It is generally accepted that as a person ages, his or her experiences acquired over the life time, ways in dealing with the environment, economic and social resources, and relationships and support systems could profoundly impact longevity and quality of life [1]. Several significant issues for researchers are (1) how to measure these constructs in an experimental design, (2) which reliable and valid measures should be employed to adequately measure these constructs, (3) how much do these psychosocial factors singly and in combination impact longevity and quality of life, and (4) how do these factors interact with other variables?

The above research questions are important and worthy for future research agendas. We hope to inform our peers on how to better understand psychosocial processes and to incorporate pertinent psychosocial predictors to enrich future longevity research. This will be accomplished by sharing a summary of how these issues are addressed in the literature in combination with recent empirical data that demonstrate their impact on health and longevity.

The goal of this paper is threefold. One, we seek to define and increase our understanding on the measurement of two commonly-used psychosocial *outcome* measures in longevity research: self-rated health and quality of life. Two, we seek to outline the impact of four generally acknowledged psychosocial *predictors* of longevity and health. These are (1) demographics, life events, and personal history, (2) personality, (3) cognition, and (4) socio-economic resources and support systems. We use a similar format in each section starting with *“What do we know?” *which is used to provide a brief introduction of the concept, followed by *“Empirical Data”* which provides published or new data from the Georgia Centenarian Study [5], a multidisciplinary population-based study of centenarians, ending with *“Impact” *which attempts to delineate the relationship of the psychosocial construct to health and well being among the oldest old. Finally, based on recent psychosocial data, we seek to update and supplement the recommendations of the 2001 NIA Panel on Longevity.

## 2. Psychosocial Outcome Measures

### 2.1. Subjective Health

Health can be measured objectively through an inventory of past and present health history, diseases, medications, hospital and physician visits, blood chemistry, and an array of biomedical markers [6]. Interestingly and surprisingly, the best predictor of overall health is the individual's perception and self-rated health [7]. The following sections outline what we know about self-related health among the oldest old and recent empirical data on the relationship between self-rated health and other subjective and objective health outcomes (e.g., functional health), other self-reported measures of health (e.g., number of hospitalizations, number of lifetime diseases, number of health problems), and biomarkers (e.g., hemoglobin and albumin) from the Georgia Centenarian Study [5].

**What Do We Know?**

A substantial amount of centenarian literature has focused on health outcomes at the limit of longevity [8–10]. The evidence is contradictory with some studies reporting high levels of frailty and morbidity among centenarians [8] whereas others reporting centenarians as being in relatively good health conditions [9, 11, 12]. In spite of the contradictory findings, there is a growing consensus that centenarians' health is a critical antecedent of well-being and quality of life in extreme old age [6, 13]. A number of studies have investigated associations involving general aspects of centenarian health such as global self-rated health [14, 15], functional health [16, 17], or the number of acute or chronic health conditions [8, 18, 19].

Of these multiple indicators of centenarian health, self-rated health might be useful to capture the multidimensionality of compounding health concerns in very old age [7, 20]. Self-rated health reflects a holistic picture of one's health and arguably may be the most meaningful dimension of health from the individual's perspective [7]. Self-rated health is a widely used health measure among oldest old adults due to its significant correlation with functioning and mortality [15, 21–23].

**Empirical Data**

As noted in the introduction, the empirical data sections in this paper are designed to discuss published or new data from the Georgia Centenarian Study. Methodological details of the study can be found in Poon, Clayton, Martin, Johnson et al. [24] and Poon, Jazwinski, Green et al. [5]. Discussion of data in this paper will focus on Phase 3 of the study (2001–2009). Phase 3 is a multidisciplinary, population-based study of 287 centenarians and near centenarians (98 yrs and older), 88 octogenarians, and 400 younger controls from 44 counties in northern Georgia. Phase 3 of the study examined genetics, neuropathology, nutrition, blood chemistry, health, diseases, medications, neuropsychology, cognition, personality, coping, distal and proximal influences, adaptation, and resources. For the present paper, we seek to focus on the two outcome variables of subjective health and quality of life as well as the four sets of predictors: life events, personality, cognition, and resources. As would be expected, the majority of the centenarian participants (85.9%) were women and White (77.4%). In terms of marital status, the majority (85.9%) was widowed, 5.6% were married, and 3.5% were divorced. A sizeable group (73.4%) had no more than a high school education whereas 15.6% had a college degree. Almost half of the centenarians resided in their private home or apartment (44.1%) whereas 19.2% resided in assisted living facilities and 36.7% in nursing facilities. Most centenarians (71.8%) reported that their health was either good or excellent. The average MMSE score of participants was 16.83. This score suggests that about half of our centenarians showed at least severe levels of cognitive impairment.

Fries et al. [25] found that all measures of physical decline increased rapidly with each year of age among the very oldest-old; hence, it should be noted that the older the elder, the lower the level of self-rated health status. Contrary to this finding, however, Liu and Zhang [23] found that the centenarians were more likely to report positively their health status but nonagenarians and octogenarians were less likely to report better health status. Empirical data from the Georgia Centenarian Study [5] seem to replicate the findings from Liu and Zhang [23]. Almost 20% of our centenarians (19.4%) rated their health as excellent, and over half of them (52.7%) rated their overall health as good. There were no significant differences in the ratings of self-reported health between centenarians and their younger counterparts (*χ* ^2^ = 2.86, *d* *f* = 3, *P* = .41). This is also consistent with Idler [26], who suggested that older subjects reported disproportionately positive health assessments, and “processes of aging, selective survivorship, and cohort differences all appear to play a role in creating this pattern (p. S289).”

 Consistent with the biopsychological approach, self-rated health showed a significant association (*P *< .05) with functional health (*r* = .32) and levels of albumin (*r *= .24) and a significant negative association with self-reported health problems (*r *= −.19). Specifically, the more health problems (e.g., chest discomfort, numbness, arthritis, and dizziness), the more dependent of physical activities in daily living (e.g., eating, dressing, walking, and bathing), and the lower the levels of albumin, which in turn lower centenarians' self-rated health.

**Impact**

Based on the above correlation results, we employed a regression model to verify the importance of health measures on self-rated health among centenarians. Significant predictors of self-rated health were number of health problems (*β* = −.14, *t* = −1.67), physical ADL (*β* = .27, *t *= 3.38), and levels of albumin (*β* = .22, *t* = 2.78) after controlling for all other variables in the model. As Table 1 shows, the model explained 17% of the variance in the centenarians' self-reported health.

### 2.2. Quality of Life, Happiness, and Well-Being

Another important outcome measure in longevity research as well as public policy is quality of life. “Adding life to years” is as important as “adding years to life” when evaluating interventions or treatment regiments among older adults. The World Health Organization [27] established a well-known definition of quality of life as “a state of complete physical, mental, and social well-being and not merely the absence of disease or infirmity” (p. 28). Contemporary social and behavioral gerontologists have traditionally conceptualized quality of life as the final criteria by which perceived resources (e.g., health, social support, socioeconomic status, cognitive functioning) influence well-being among centenarians [28–30] whereas clinical geriatric investigators have generally operationalized quality of life as contributing health-related indicators (e.g., biomarkers, type of acute illness or chronic disease, performance of activities of daily living, family health history) of disability and mortality in longevity [12, 19, 31, 32]. Nonetheless, there is consensus that quality of life reflects health related as well as nonhealth associated indicators [33]. A biopsychosocial approach may provide an effective alternative in differentiating quality of life among persons of exceptional old age.

**What Do We Know?**

Although quality of life, happiness, and well-being denote similar constructs, psychologists have attempted to differentiate these constructs in subtle ways. Ryff [34] noted that perceptions surrounding quality of life, happiness, and well-being are in the eye of the beholder. Researchers often have the task of deciding whether to assess objective components (e.g., physical biomarkers or activities of daily living performance) or subjective characteristics (e.g., self-reported ratings) of well-being [33].

Happiness is often used as an outcome variable and is broadly defined as an individual's state of being [35]. Happiness is theorized to be a positive affective condition which arises from the comparison of former life accomplishments and achievements to current life situations and circumstances (e.g., health status, social support, economic status [35]). Diener and Biswas-Diener [36] have referred to this mechanism as psychological wealth. In other words, happiness is indicative of the degree to which persons feel they have effectively used resources in the past, yet remain optimistic and feel they still have enough to flourish in the present or future. Diener and Biswas-Diener [36] integrated this perspective into a common formula

(1) $$\text{Happiness}=\frac{\text{What}\;\text{we}\;\text{have}\;\left(\text{attainments}\;\text{of}\;\text{life}\right)}{\text{What}\;\text{we}\;\text{want}\;\left(\text{current}\;\text{aspirations}\right)}.$$

From an evolutionary psychology perspective, happiness signifies the achievement of successful adaptation and expert survivorship in aging [37]. Happiness is achieved when appropriate steps are taken to attain acceptance and fulfillment with one's past (i.e., congruence) as well as to effectively use resources to solve everyday problems which threaten health, well-being, and survival [37]. Perhaps, happiness is most optimal among persons who have survived beyond advanced old age.

Older adults reaching advanced old age often experience physical inactivity, poor nutritional health, anxiety, and greater feelings of fatigue [38]. In turn, our work has focused on examining how perceived contentment with the past and psychosocial resources may bolster or diminish perceived well-being or happiness. We reported health impairment as a key indicator and mediating variable of congruence (e.g., perceived degree of contentment with the past) across a sample of old and very old adults [26]. In particular, social support and socioeconomic status (SES) had indirect associations on congruence and happiness through health impairment. Furthermore, we reported that social support, SES, health impairment, and congruence (e.g., degree of contentment with the past) explained 40% of the variance in happiness.

**Empirical Data**

In an investigation involving 158 centenarians, we were interested in understanding how perceived degree of available psychosocial resources would mediate the relationship between congruence and happiness [39]. Based on our previous work, it was hypothesized that perceived health status would emerge as a key indicator and mediating variable of happiness. However, there was no significant evidence of mediation. Instead, congruence emerged as a key predictor of how centenarians perceive resources and determinant of feeling happy. Greater satisfaction with the past had a significant direct association with perceived economic security, perceived health status, and current happiness. Forty-four percent of the variance in perceived health was explained by congruence, economic security, and social provisions. In addition, 25% of the variance in happiness was explained by congruence, perceived economic security, social provisions, and perceived health status.

**Impact**

Table 2 provides a summary of our most recent findings from the Georgia Centenarian Study using positive and negative effect as key mediating influences between health and psychosocial indicators and happiness among centenarians. Results confirm that positive emotionality is associated with greater feelings of happiness whereas negative effect diminishes happiness. Fatigue appears to be a salient predictor of positive and negative effect. Fatigue had a significant negative direct effect on positive emotionality but a significant positive direct effect on negative effect.

## 3. Psychosocial Predictors

As noted earlier, there is no argument that an individual's personal history, known as distal influences, and their current conditions, known as proximal influences, could combine to impact longevity and quality of life [1, 40]. The critical questions are which distal and proximal influences are most influential in determining longevity and quality of life and how do we investigate their direct and indirect impacts [40]. In this section, we review four selected psychosocial areas that have been found to influence longevity and quality life. 

### 3.1. Demographics, Life Events, and Personal History

Life events are important determinants of physical and mental health in older adults, especially stressful events [41, 42]. Life events are experiences that can have a positive/negative and proximal/distal influence on centenarians' health and quality of life. Studying the effect of life events on the oldest-old is important because they most likely have experienced a significant number of positive and negative events over their life time. Centenarians may have experienced events such as decline in health, loss of loved ones, institutionalization, and even deterioration of their financial resources. Therefore, the events that many centenarians have experienced and have been exposed to may have impacted their current physical and mental health status as well as influenced their survivorship and overall quality of life.

**What Do We Know?**

Life events can play a role in human development as both distal and proximal influences [40]. The literature provides support that distal factors could clearly impact health and quality of life in old age. For example, one of the most crucial studies published on this topic involves the work of Blackwell et al. [43]. In their research, they examined whether childhood health had long-term and enduring consequences for chronic morbidity. Results indicated that poor childhood health increases morbidity in later life, and that this correlation was found for cancer, lung disease, cardiovascular conditions, and arthritis. Childhood health was a key factor in the risk for a heart attack in later life in a study conducted by O'Rand and Hamil-Luker [44]. These researchers investigated how cumulative adversity across the lifespan influenced risk trajectory for heart attack. Their results suggested that early disadvantage and childhood illness have immense enduring effects and do increase the risk for heart attack in later life. However, adult pathways influence these risk trajectories and mediate the effects of early disadvantage. Building on the work of O' Rand and Hamil-Luker [44] and others, McEniry et al. [45] studied the influence of early life exposure to poor nutrition and infectious diseases on the health of older Puerto Ricans. These researchers found a strong association between exposure and heart disease, and a weaker association between exposure and diabetes. They concluded that the timing of birth is associated with conditions occurring around the time of birth that can affect heart disease and diabetes in later life. Distal influences and their effect on cognition in later life was the focus of a study done by Fors et al. [46]. This investigation looked at the association between childhood living conditions, socioeconomic position in adulthood, and cognition in later life. Results showed that exposure to conflicts during childhood, having a father classified as manual worker, low education, and/or being classified as a manual worker in adulthood was associated with lower levels of cognition in old age. Besides childhood health and living conditions, life events are also of interest to gerontologists in terms of their influence in lifespan development.

**Empirical Data**

In the Georgia Centenarian Study, we assessed events that occurred over the life span such as marriage, birth of a child, death of spouse, child, and siblings, health events, historical events, retirement, personal injury, worsening relationship with child, and institutionalization. Investigating the proportion of some of these life events experienced by centenarians at any time of their lives, we found that 87.3% of centenarians had experienced the death of a spouse and 32.1% had experienced the death of children. Centenarians reported a high proportion of decline in activities (89.9%) and hospitalization (97.8%).

When comparing centenarians' mean scores on several different measures of life events to octogenarians we concluded that centenarians reported significantly lower mean scores on number of proximal and positive events compared to octogenarians. In contrast, centenarians reported a higher mean score on number of distal events (Table 3).

Several distal influences were found to impact the health of centenarians from the Georgia Centenarian Study [47]. The data show that the number of children significantly predicted centenarian's ability to engage in activities of daily living (*β* = .14, *P *< .05) and loneliness (*β* = −.23, *P* < .05). In essence, the more children, the higher the activities of daily living score and the lower the loneliness scores. Family history variables could account for 12% of variance in loneliness. Moreover, distal variables also influenced physical health in centenarians [47]. First, childhood health significantly predicted current health problems (*β* = −.233, *P* < .05). The poorer one's health in childhood, the larger the amount of current health problems reported. In this investigation, family history variables accounted for 13% of the variance in current physical health problems. Second, lifetime negative events significantly predicted current health problems (*β* = .33, *P* < .01). The more life-time negative events, the greater the amount of current health problems reported. In these findings, life events accounted for 10% of the variance in current physical health problems. These results confirm the importance of distal family history variables (influences) on present-day functioning of older adults.

**Impact**

Looking at the relationship between positive/negative life events and self-rated health, we conclude that cognitively intact centenarians that experienced a high number of negative events reported lower scores on self-rated health (*r* = −.21, *P *< .05). In contradiction, positive life events was positively associated with self-rated health (*r *= .19, *P *< .05). In addition, a high number of negative events was negatively associated with lower levels of congruence (i.e., contentment with past achievements) (*r *= −.27, *P *< .01) and life satisfaction (*r *= −.21, *P *< .05), suggesting that centenarians with a high number of negative events in life rated lower levels of contentment with past achievements and life satisfaction. In addition, individuals who experienced a high number of total events had lower levels of contentment with their past achievements (*r* = −.19, *P *< .01). Drawing on the material presented, distal variables do indeed influence functional, mental, and physical health in the oldest old. Not only does evidence exist for the number of children affecting both activities of daily living and loneliness in older adults [47], but childhood health influences current health problems in centenarians.

### 3.2. Personality

When facing life stress or life changes, centenarians (like other age groups) draw on a number of different resources. One important individual resource centenarians can draw on is their own personality. Although many aspects in very late life may change, centenarians can rely on a set of personality traits that help with everyday problems. A personality trait approach is typically used to describe personality profiles of centenarians.

**What Do We Know?**

Several studies have assessed personality traits of centenarians [48]. A Swedish centenarian study [49], for example, suggested that centenarians could be described as dependable, reliable, mature, conscientious, and less frequently participating in social activities. Furthermore, centenarians on average were responsible, easygoing, capable, relaxed, efficient, and not prone to anxiety. Results from the first Georgia Centenarian Study [24] indicated that centenarians had higher scores in dominance, suspiciousness, and shrewdness whereas they were lower in imagination and tension when compared to two younger groups [50]. When retesting centenarians after approximately 20 months, we found that centenarians had decreased scores in sensitivity, but higher scores in radicalism [51]. Martin [50] suggested that the “robust personality” among these highly selected centenarians was a possible indicator of survivorship but also an important resource that may help centenarians adapt to everyday problems in very late life. Findings from the most recent Georgia Centenarian Study using the Big-5 framework confirmed a unique set of robust personality traits including low levels of neuroticism, but high levels of extraversion, conscientiousness, and agreeableness [38]. These results confirmed that centenarians showed several unique traits, but that a special combination of traits (i.e., low levels of neuroticism, high conscientiousness, and moderately high extraversion) were also notable in this group of exceptional survivors [38].

Low levels of neuroticism in female centenarians as measured by the NEO Big-5 were also reported by Silver et al. [52] and a recent Japanese centenarian study indicated that male and female centenarians scored higher in openness. Another Japanese centenarian study reported that centenarians had high scores in femininity and low scores in Type-A behavior [53]. 

In summary, personality traits are usually included in centenarian studies because of their possible contribution to longevity and adaptation. The most consistent personality trait found in almost every centenarian study focused on low levels in Neuroticism [48].

**Empirical Data**

Although there is quite a bit of research on personality traits, their relationship to health and quality of life outcome measures is still not thoroughly investigated for centenarians. Are centenarians with low scores in neuroticism also healthier? Does extraversion improve quality of life? Table 4 summarizes our most recent findings using the Big-5 framework (i.e., neuroticism, extraversion, openness, agreeableness, and conscientiousness) to predict two different quality of life outcomes: mental health and subjective health. All personality trait ratings of centenarians were provided by proxy informants.

The results of two separate multiple regression analyses indicate that three of the five personality dimensions were significantly associated with mental and physical health measures. Conscientiousness was a consistent predictor: centenarians who were rated as more conscientious were reported to be in better health, but had lower scores in mental health. In addition, neuroticism was negatively and openness positively related to mental health.

**Impact**

Whether centenarians achieve high levels of quality of life or not may depend on many psychosocial or biological factors. However, personality traits may directly indicate why life for some centenarians is still as enjoyable and autonomous as it has been in their earlier years. The “robustness” of centenarians, as indicated by low levels of neuroticism and relatively high levels of openness to experience and especially conscientiousness, helps us understand individual differences in late life adaptation.

### 3.3. Cognitive Functioning

The 2001 NIA Panel on Longevity acknowledged that cognitive functioning is an important predictor to be included in the study of human longevity. It is noted that cognition is multi-dimensional consisting of both global measures and specific cognitive and neuropsychological mechanisms. Key decisions that confront longevity researchers in the design of longevity studies are which cognitive measures should be included and how comprehensive the cognitive battery should be [54]. While there are different schools of thought in response to these questions, the general rule of thumb is the selection needs to be based on the specific aims and hypotheses of the research [55]. The goal of this section is to demonstrate that cognition is highly correlated with other psychosocial variables as well as indicators of mental and physical health in determining quality of life among the oldest old.

**What Do We Know?**

Cognitive functioning is a predictor of critical outcomes in older age including institutionalization, everyday functioning, and longevity [56]. Several factors are related to the achievement and maintenance of cognitive functioning and vitality among centenarians including social support, physical health, nutrition, personality, and mental health (for discussion see Margrett et al. [57]. Cognitive abilities work in concert with such factors to shape older adults' quality of life. Unfortunately most empirical work examining cognition in older adulthood relies on participants who represent the young old (i.e., 65–75) or old old (i.e., 75–84), thereby truncating the ability to examine the full developmental course of cognitive aging. Thus, centenarian studies are critical to improving knowledge regarding cognition and the role it plays, together with other skills and resources, in promoting longevity and successful aging. There are several issues of theoretical and methodological importance that should be noted when examining cognitive functioning in late life. One issue is the impact of physical and sensory limitations on test results. As discussed by Holtsberg et al. [58] an increased prevalence of sensory and mobility limitations can result in automatic deductions from tests such as the MMSE which includes items relying on visual cues (e.g., read and follow directions, copy write a sentence). For majority populations, the general cut-off for the MMSE is 23, with scores lower than 23 indicating likely impairment. For samples varying in ethnicity, education, and degree of sensory impairment or disability, this cut-off has been questioned resulting in use of an adjusted or lower cut-off score (i.e., 17, 21) [59, 60]. Many cognitive tests lack normative data, particularly for the oldest old. This can make group comparisons difficult (e.g., cohort differences in education) [58] as well as hamper interpretation of individual performance. As a result, researchers should carefully consider appropriate cut-off scores for centenarians and the oldest old.

A second issue related to investigation of cognition in late life is assessment choice. Cognitive measures vary along three related dimensions: (a) focus on normative age-related cognitive change in the context of understanding impaired functioning associated with dementia; (b) level of specificity ranging from global assessments of status (e.g., MMSE) to specific tests of particular cognitive abilities (e.g., episodic memory); and (c) degree of emphasis on the application of cognitive abilities to everyday life which can indicate probable day-to-day functioning and impairment. Heterogeneity of cognitive outcomes increases with age and the prevalence of dementia is greater among the oldest old (37%–50%). The possible prevalence approaches 80% among centenarians [56, 61, 62]. Owing to participant fatigue and potentially diminished cognitive capacity, multiple sources (e.g., self-report, proxy-ratings, interviewer observations, performance-based tests) are often included in studies of the oldest old. Recent analyses of the Georgia Centenarian Study demonstrate how differences in data sources can lead to varying interpretations [63].

**Empirical Data**

The assessment strategy employed in the Georgia Centenarian Study reflects the multidimensionality of cognition. The major hypotheses focused on the prevalence of dementia in a population-based sample as well as the identification of cognitive mechanisms associated with everyday functioning abilities among the oldest old. As is typical in many gerontological studies, cognitive or mental status was initially assessed by the Mini-Mental Status Exam (MMSE) [64]. We further included a clinician assessment scale, the Global Deterioration Scale [65, 66], to complement the MMSE. However, other more comprehensive measures were utilized including indicators of: (a) global cognitive ability (Severe Impairment Battery, SIB) [67], (b) executive control (Behavioral Dyscontrol Scale, BDS) [68], (c) verbal fluency (Controlled Oral Word Association Test, COWAT) [69], (d) memory (Fuld Object-Memory Exam, FOME) [70], and (e) language abilities (Similarities subtest from the WAIS-III) [71].

In the following analyses of data from the Georgia Centenarian Study, we first address the range of cognitive functioning among centenarians and second we compare the utility of several indicators of cognitive functioning in predicting centenarians' self-rated mental and physical health. Prior research suggests that cognitive abilities may become more similar, or dedifferentiated [72] resulting in high correlations between measures. This hypothesis has not been fully explored among the oldest old, although work is underway using the GCS data. In the present study, the selected cognitive measures were strongly correlated among centenarians overall (i.e., range = .44–.87). Thus, we conducted separate multiple regression analyses to examine the utility of each cognitive measure in predicting subjective mental and physical health.

To address the first question, we examined MMSE performance among octogenarians and centenarians. While 85% of octogenarians scored 23 or greater, centenarians demonstrated greater diversity in performance with 32% achieving the same score. The use of multiple cutoff scores illustrates the importance of considering appropriate cutoff scores when assessing the cognitive functioning of very old individuals; our data suggest that 68% of centenarians would likely be classified as impaired using a traditional cutoff score of 23 on the MMSE. However, given this group's sensory impairments, educational level, and ethnic diversity such a conclusion may not be warranted. To illustrate, we recently published normative data from the GCS on several measures, including the MMSE [73]. In this case, at the point of this oldest old age group, MMSE scores declined on a nearly yearly rate (i.e., 50th%ile at 98-99 yo = 21; 100-101 yo = 16; 102+ yo = 13). As we recently argued [57], the entire range of cognitive functioning must be considered in order to appreciate the full spectrum of cognitive health in later life.

Table 5 depicts results of the regression analyses examining individual cognitive predictors of centenarians' health. Perhaps not surprising given the age-related physical limitations faced by many centenarians, cognitive measures were better predictors of mental health as compared to physical health accounting for a greater proportion of the explained variance. The analyses examining physical health revealed that COWAT performance was a significant predictor, as well as a trend for the WAIS-III Similarities subtest. For mental health, the MMSE, COWAT, and WAIS-III Similarities subtest (*P* < .10) were each significant predictors within the separate models above and beyond the effects of sex, ethnicity, residential status, and education. This finding suggests that cognitive status, abstract reasoning, and capacity for quickly producing verbal responses are important contributors to mental health, perhaps in the context of interpersonal relations. These regression analyses indicate the differential utility of cognitive assessments.

**Impact**

Because there have been no normative data for centenarians, the relative utility of any particular cognitive instrument is difficult to place into context. We found that the MMSE is probably sufficient to ascertain an overall level of cognitive functioning, and the MMSE predicts most of the variance in basic and instrumental activities of daily living (BADL and IADL). More specific neuropsychological instruments are somewhat time consuming to administer, but they are equally as predictive (as a group) as the MMSE of BADL and IADL. However, they are able to yield more domain-specific information about an individual's cognitive functioning. The extant literature, as well as recent findings from the Georgia Centenarian Study, demonstrate that cognitive abilities work in conjunction with other psychosocial variables as well as indicators of physical health in determining quality of life among the oldest old. It is also clear that generalizations across age groups can be misleading as differences can be profound between individuals classified as the “oldest old,” as seen in differences between octogenarians and centenarians in this sample. These findings can inform prevention and intervention efforts, which would benefit from: (1) a more multi-dimensional, holistic approach and (2) targeting potential mediating factors earlier in the life course.

### 3.4. Social and Economic Resource Adequacy

Our final psychosocial predictor of centenarian longevity is based on the construct of resource adequacy, which includes dimensions of both economic resources and social resources. For this study, using the Duke OARS [74] we obtained Georgia centenarians' perceptions of the adequacy of their economic (i.e., perceived economic status) and social resources. In addition, we used Cutrona and Russell's [75] Social Provisions Scale which included various aspects of the quality of social relationships.

**What Do We Know?**

About a decade ago, gerontologists began to describe centenarians' social networks and interactions [76, 77] as well as their economic resources [78]. A few centenarian studies began to establish the association of these resources to life satisfaction [79] and mental health [77]. Poon and colleagues [24] found that there was a significant bivariate association between social resources and Georgia centenarians' longevity. Martin [50] applied the Georgia resource model of developmental adaptation to predict centenarians' mental health and functional health, testing whether adverse and more distal cumulative life events would have direct, as well as indirect effects mediated by proximal social support and economic resources. Martin [50] found that adverse cumulative life events reduced both social resources and perceived economic status; both types of resources were positively related to mental health. In addition, it is understood that a centenarian's residential setting (e.g., private home, skilled nursing facility, or nursing home) is associated with these measures of social resource adequacy [80] and that the amount and type of services obtained from paid or unpaid caregivers are related to their physical and mental health [81]. The research reported here builds upon these studies to assess the impact of social and economic resource adequacy on centenarians' self-reported mental health and physical health in the Georgia Centenarian Study.

Randall et al. [80] found evidence of age-related social resource decline; centenarians self-reported significantly lower levels of social resources and social provisions than octogenarians. MacDonald et al. [81] found that centenarians also used more types of caregiving services than octogenarians, but there were no significant age group differences with respect to total caregiving hours, income support, or medical care usage. However there was a significant positive relationship between caregiving hours received and the personality trait of competence, controlling for residential setting, and perceived health status. MacDonald and colleagues [63] also analyzed the influence of perceived economic status on alternative perceptions of centenarians' mental health by comparing multivariate single-regression models predicting self-, proxy, and interviewer reports. There was a significant positive relationship of perceived economic status to both self- and proxy mental health reports (controlling for nursing home residence, distal engaged lifestyle and cumulative distal events, ADL, subjective health, and mental status). Recently, Randall et al. [82] also reported positive relationships of both self-reported perceived economic status and social resources with the outcome, centenarians' mental health (interviewer report), that were obtained from structural equation models including distal manifest variables (childhood socio-economic status, and stressful life events experienced at various ages) as exogenous predictors and intervening proximal latent economic and social resource variables. A single item measure of self-rated physical health as a summary assessment of overall health status has been validated in the literature and found predictive of outcomes such as mortality, BMI, physical activity, and hospitalization among others [83–85]. DeSalvo and colleagues [83] compared the predictive accuracy of a single-item measure of general health with multi-item scales (e.g., mental component summary and physical component summary). The single item performed as well as the multi-item measures regarding validity and reliability, in addition to saving time and money over the use of longer instruments. Similarly, studies have assessed single-item self reports of mental health. However, when possible it has been suggested to use multiple items to assess the breadth associated with such a construct as mental health [86].

**Empirical Data**

The present study, using data from the Georgia Centenarian Study, investigated predictors of centenarians' self-reported mental health (a summed index of the twenty OARS Mental Health self-reported items) and we used the same model to predict centenarians' self-reported physical health. This allowed us to compare how these predictors differentially were associated with key health and quality of life outcomes.

First, for the self-rated mental health outcome, we specified a hierarchical regression model with three blocks of predictors. (a) Control variables: residential type, sex, cognitive status, ethnicity, and education; (b) health correlates: functional and physical health; and (c) resource adequacy measures: perceived economic status, social provisions (an instrumental assessment of social support), and social resources (a structural assessment of social support). We present results from the final model including statistics for the health and socio-economic resource adequacy predictors (Table 6).

In the final model, two of the variables included in the third block of socio-economic adequacy resource measures were significantly associated with the outcome, mental health, as this block also significantly contributed to the increase in variance explained (*F *Δ = 5.73; *P* = .001). Perceived economic status and social resources significantly predicted mental health; only social provisions did not contribute to an increase in centenarians' mental health controlling for all other variables in the model. As Table 6 shows, the final model explained 56% of the variance in the centenarians' self-reported mental health. The addition of the socio-economic resource adequacy predictors in the final block increased the variance explained by over 17% relative to the model that excluded those predictors. 

Next, we investigated the same model predicting self-rated health. One intriguing finding was noted in the final model that explained 37% of the variance: social provisions significantly predicted physical health controlling for all other predictors in the total model. Thus, it appears that for centenarians, the type of social support provided is significantly related to their self-rated health whereas their perception of economic sufficiency and their amount of network contact do not.

**Impact**

As Antonucci et al. [87] theorized, assessments of social, economic, and personal resources significantly contribute to self-rated mental and physical health in advanced old age beyond the contribution of various controls (e.g., sex, race, residential setting, cognitive status, and education) and functional health.

## 4. Conclusions and Recommendations

The goal of this paper is to highlight the importance of psychosocial factors on longevity research. We have shown that a selected number of psychosocial predictors and outcome variables could provide a comprehensive picture of health, functioning, and quality of life at extreme old age. Inclusion of these variables along with biomedical mechanisms could enrich our understanding on what, how, and why some individuals survive to a ripe old age with high quality of life while others could not.

There is much to be done to better understand psychosocial contributors to longevity and the psychosocial and biomedical mechanisms that combine and interact to increase longevity and quality of life. It is worthwhile to note that experiences from seasoned centenarian researchers [1] cautioned us to pay particular attention to measurement fatigue among centenarians, issues of reliability and validity of short forms of tests, suitable use of criterion or cut-off scores owing to compromise sensory and cognitive functions [58], as well as the validity of using proxy when direct measures are not available.

The final goal of this paper is to supplement the *2001 NIA Panel on the characterization of participants in studies of exceptional survival in humans* with recommendations based on empirical psychosocial data associated with health and quality of life among the oldest old.

1. Self-reported or subjective health serves as a useful index of physical trajectories of current health status as well as an indicator of the presence or absence of resources that might influence functional decline. Self-rated health shows a significant association with functional health (*r* = .32) and levels of albumin (*r *= .24) and a significant negative association with self-reported health problems (*r *= −.19).
2. Quality of life, happiness, and well-being are important public health and public policy outcomes in the evaluation of intervention and treatments at the end of life. Care must be exercised in measuring these variables as positive and negative emotions and effects are associated with fatigue, functional health, and cognition.
3. Information of distal life events and recent proximal influences provides meaningful information about the aged individual. A high number of negative events correlate with lower scores on self-rated health (*r *= −.21, *P *< .10), while positive life events are positively associated with self-rated health (*r *= .19, *P *< .10). In addition, a high number of negative events are negatively associated with lower levels of congruence (contentment with past achievements),* r *= −.27, *P *< .10 and life satisfaction (*r *= −.21, *P *< .10), suggesting that centenarians with a high number of negative events in life report lower levels of contentment with past achievements and life satisfaction.
4. As noted by the 2001 NIA Panel, we concur that an individual's personality, as measured by the Big-5 personality traits, could impact longevity and health. In addition, we have found that three of the five personality dimensions are significantly associated with mental and physical health measures. Conscientiousness is a consistent predictor. Centenarians who are rated as more conscientious are reported to be in better health, but had lower scores in mental health. In addition, neuroticism is negatively and openness positively related to mental health.
5. While the 2001 NIA Panel endorsed the measure of cognition as important to longevity, institutionalization, mental and physical health, we caution the need to include sensory capacities, literacy, and the choice and criteria in the selection of the cognitive instrument that is suitable to the sampled individuals as well as everyday functions.
6. Social and economic resources in conjunction with functional and physical health could account for 56% of self-rated mental health and 37% of self-rated physical health. Social and economic resource adequacy are recommended as pertinent variables for better understanding health and longevity with particular relevance for public policy.

Evaluating comprehensive quality of life domains among centenarians is important. After all, life would only be worth extending to a second century if it came with a minimum level of health, autonomy, and functioning. However, focusing exclusively on health aspects would disregard the importance of a number of psychosocial domains, including life events, personality, cognition, and social supports, that are also essential for a rewarding life at 100 and beyond. The relative impact and significance of these domains are, of course, dependent on the research question. We conclude that these psychosocial domains are as important and have the highest potential to interact with biological and medical aspects in unearthing the secrets to exceptional longevity.

## Figures and Tables

**Table 1:** Predictors of centenarians' self-rated health.

Predictor variables	B	SE	*β*
Past diseases	−0.02	0.06	−0.03
Current diseases	−0.00	0.07	−0.01
No. of health problems	−0.04	0.02	−0.14^†^
No. of hospitalization	−0.00	0.03	−0.00
Physical ADL	0.08	0.02	0.27**
Albumin	0.49	0.17	0.22**
Hemoglobin	−0.05	0.04	−0.09
*F*	4.21***		
*R* ^2^	0.17		

^†^ *P* < .10. **P < .01. ***P < .001.

**Table 2:** Positive and negative effect and happiness.

Variable (Model 1)	Direct effect	SE	Indirect effect	Total effect	*r*
Positive effect					
Perceived health	.17	.27	.04	.21	.31
Functional health	−.12	.06	−.03	−.15	.13
Cognition	.13	.11	.03	.16	.22
Fatigue	−.41**	.04	−.10	−.51	−.45
Distal events	.12	.17	.03	.09	.14
Happiness					
Positive effect	.24*	.09	—	—	.04

Variable (Model 2)	Direct effect	SE	Indirect effect	Total effect	*r*

Negative effect					
Perceived health	−.17	.16	.06	−.11	−.20
Functional health	.26*	.08	−.09	.17	-.08
Cognition	−.26*	.15	−.09	.35	−.21
Fatigue	.26**	.03	−.08	.18	.27
Distal events	.10	.10	.03	−.07	−.10
Happiness					
Negative effect	−.33**	.12	—	—	−.09

*Note.* Indirect effects calculated by multiplying direct effects between psychosocial indicators and positive and negative effects with the direct effects between positive and negative affect and happiness. Total effects equal sum of direct and indirect effects. Dashed lines indicate no calculation of indirect or total effect.

*P< .05, **P< .01.

**Table 3:** Age group comparisons on life events.

	Centenarians (*n *= 137)		Octogenarians (*n *= 71)		*F*
	Mean	SD	Mean	SD	
Number of proximal events	2.14	1.39	3.70	1.69	50.76***
Number of distal events	5.07	1.62	4.38	1.37	9.51**
Total number of life events	11.76	1.98	11.62	1.81	.25
Positive life events	4.28	1.44	5.27	1.21	24.25***
Negative life events	7.44	2.22	7.01	2.07	1.78

*P < .10, **P < .05, ***P < .001.

**Table 4:** Personality predictors of centenarians' self-rated mental health and physical health.

	Mental health			Physical health		
	B	SE	*β*	B	SE	*β*
Functional capacity	.19	.06	.28**	.04	.01	.40***
Subjective health	2.12	.43	.33***	x	x	x
Mental health	x	x	x	.06	.01	.39***
Neuroticism	−.08	.02	−.34***	.00	.00	−.06
Extraversion	.02	.02	.08	.00	.00	−.08
Openness	.05	.02	.16∗	.00	.00	−.08
Agreeableness	.00	.02	.01	.00	.00	−.11
Conscientiousness	−.04	.02	−.19*	.01	.00	.20*
Model *R* ^2^			.48			.39

Models controlled for sex, ethnicity, cognitive status, residential type, and education.

*P < .05. **P < .01. ***P < .001. (two-tailed tests).

**Table 5:** Cognitive predictors of centenarians' self-rated mental and physical health.

Predictors	Mental health				Physical health			
	B	SE	*β*	*R* ^2^	B	SE	*β*	*R* ^2^
Mini-Mental Status Exam	.26	.09	.26*	.13*	.02	.02	.12	.02
Global Deterioration Scale	−.63	.40	−.16	.11	−.04	.07	−.06	.03
Severe Impairment Battery	.08	.06	.14	.10	-.01	.01	−.09	.03
Behavioral Dyscontrol Scale	.12	.11	.11	.09	.02	.02	.12	.07
COWAT	.35	.13	.27*	.15*	.06	.03	.25*	.08*
FOME retention	−.03	.18	−.02	.09	−.05	.03	−.15	.04
WAIS-III Similarities subtest	.11	.06	.22^t^	.12^t^	.02	.01	.21^†^	.05^†^

Models controlled for sex, ethnicity, residential status, and education.

^†^ *P* < .10. *P < .05. (two-tailed tests).

**Table 6:** Predictors of centenarians' self-rated mental and physical health.

Predictors	Mental health			Physical health		
	B	SE	*β*	B	SE	*β*
Functional health	.27	.07	.30***	.01	.02	.02
Physical health	2.01	.47	.35***	x	x	x
Mental health	x	x	x	.09	.02	.51***
Perceived economic status	.7	.23	.25**	.03	.05	.06
Social provisions	−.07	.15	−.03	.06	.03	.17*
Social resources	.61	.22	.21**	.03	.05	.05

Model *R* ^2^			.56			.37

Models controlled for sex, ethnicity, cognitive status, residential type, and education.

*P < .05. **P < .01. ***P < .001. (two-tailed tests).
